# Effects of paternal overnutrition and interventions on future generations

**DOI:** 10.1038/s41366-021-01042-7

**Published:** 2022-01-12

**Authors:** Md Mustahsan Billah, Saroj Khatiwada, Margaret J. Morris, Christopher A. Maloney

**Affiliations:** 1 School of Medical Sciences, UNSW Sydney, Sydney, NSW 2052 Australia; 2 School of Health Sciences, UNSW Sydney, Sydney, NSW 2052 Australia

**Keywords:** Diseases, Medical research

## Abstract

In the last two decades, evidence from human and animal studies suggests that paternal obesity around the time of conception can have adverse effects on offspring health through developmental programming. This may make significant contributions to the current epidemic of obesity and related metabolic and reproductive complications like diabetes, cardiovascular disease, and subfertility/infertility. To date, changes in seminal fluid composition, sperm DNA methylation, histone composition, small non-coding RNAs, and sperm DNA damage have been proposed as potential underpinning mechanism to program offspring health. In this review, we discuss current human and rodent evidence on the impact of paternal obesity/overnutrition on offspring health, followed by the proposed mechanisms, with a focus on sperm DNA damage underpinning paternal programming. We also summarize the different intervention strategies implemented to minimize effects of paternal obesity. Upon critical review of literature, we find that obesity-induced altered sperm quality in father is linked with compromised offspring health. Paternal exercise intervention before conception has been shown to improve metabolic health. Further work to explore the mechanisms underlying benefits of paternal exercise on offspring are warranted. Conversion to healthy diets and micronutrient supplementation during pre-conception have shown some positive impacts towards minimizing the impact of paternal obesity on offspring. Pharmacological approaches e.g., metformin are also being applied. Thus, interventions in the obese father may ameliorate the potential detrimental impacts of paternal obesity on offspring.

## Introduction

The global rise in obesity has become a major health challenge due to increased risk of chronic diseases such as type 2 diabetes mellitus (T2DM), cardiovascular disease (CVD), kidney disease, cancer, and infertility thereby decreasing quality of life across the world [1]. Genetic factors, decreased physical activity, stress and environmental factors (e.g., availability of energy dense foods, endocrine disrupting compounds such as bisphenol A, dichlorodiphenyltrichloroethane) are considered risk factors for the current epidemic [2, 3]. Recently parental pre-conceptional/peri-conceptional exposure to overnutrition has been found to increase the risk of obesity and associated metabolic and reproductive disorders in offspring, independent of genetic makeup [4, 5]. While maternal health status and nutrition during gestation and lactation have a larger contribution than the father’s on offspring health, paternal influences cannot be neglected [6]. Emerging evidence shows that paternal obesity can have substantial negative impacts on the metabolic and reproductive health of offspring [7, 8]. Recent evidence shows that interventions in obese fathers can reduce the negative impact of paternal obesity on offspring [9–11]. In this review, we examine recent studies on the effects of paternal obesity on offspring health, including the proposed mechanisms, particularly the role of sperm DNA damage. We also examine studies on paternal interventions, particularly exercise, dietary modification, micronutrient supplementation and glucose lowering drug which have been found to offer promise in minimizing the adverse effects of paternal obesity on offspring.

## Developmental origins of health and disease (DOHaD)

“Developmental origins of health and disease” (DOHaD) or simply “developmental programming” refers to a permanent alteration in the physiology, metabolism, and epigenome of an offspring by the exposures (e.g., overnutrition, undernutrition, smoking) of that offspring’s father or mother before conception or during gestation or exposures during an early stage of that offspring’s life, which increase susceptibility to disease in adulthood of that offspring [4, 12]. Hence, the “DOHaD” concept links the state of health and disease risk in adult life with the environmental conditions during early life (i.e., conception, pregnancy, infancy, childhood adolescence, and early adulthood) [13]. This concept evolved from the “Barker hypothesis” postulated by Barker [14] and the “Thrifty phenotype hypothesis” proposed by Hales and Barker [15]. Both hypotheses primarily focused on how nutritional status of a mother during gestation could program offspring health. Very recent reviews of the evidence to date propose that both paternal and maternal pre-conceptional health is critical for future health outcomes [4, 5]. Our review will particularly focus on the effects of paternal pre-conception state on offspring health outcomes since these outcomes have been relatively less well documented than maternal programming.

## Paternal origins of health and disease (POHaD)

**Fig. 1:**
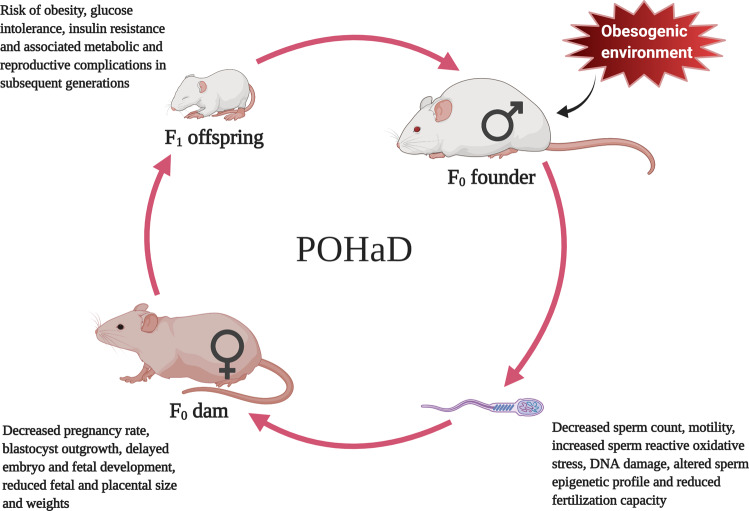
Schematic demonstration of paternal origins of health and disease (POHaD) (created with BioRender.com). Obesogenic environment (e.g., high caloric diet, sedentary lifestyle) can compromise father’s sperm quality (increase sperm oxidative DNA damage, increase sperm epigenetic modification, reduce fertilization capacity) which may have substantial negative impacts on embryo and fetal development, thus predisposing the future generation to metabolic and reproductive complications. Thus, a perpetuating cycle can ensue.

Emerging evidence from both human and rodent studies suggests that a father’s pre-conceptional health status may have a critical impact on embryo development (see review [16]), as well as the metabolic and reproductive health of future generations (see Tables 1–3). Table 1 summarizes the relevant human studies of paternal programming effects on offspring while Tables 2 and 3 summarize relevant animal studies of obesity-induced paternal programming effects on offspring metabolic and reproductive health respectively. With such evidence, a new concept “Paternal Origins of Health and Disease” (POHaD) was introduced by Houfflyn et al to stress the potential role of paternal pre-conceptional exposures in passing current environmental information to their future generations [8]. Hence, the paternal environment should not be overlooked. The basic concept of developmental programming induced by paternal obesity is illustrated in Fig. 1.

**Table 1:** Relevant human studies showing paternal programming of offspring and/or grand-offspring health.

Reference	Paternal (F0) factor	Affected generation	Sample size	Study name	Outcomes	Cohort country
[32]	Food availability (prenatal)	F_1_	360 adult offspring (mean age 37 years) and their parents.	DFBC	Adult offspring: ↑ bodyweight (+4.9 kg) and ↑ BMI (+1.6 kg/m^2^) if their fathers were prenatally exposed to undernutrition.	Netherland
[33]	Birthweight	F_1_	3659 fathers and 662 LGA infants.	SCOPE	Fathers of LGA infants: 180 g heavier at birth compared to fathers of non-LGA infants.	Australia, Ireland, New Zealand and UK
					LGA: birthweight >90th centile as per “Intergrowth 21st standards”	
[34]	Birthweight	F_1_	2002 couples and their infants.	SCOPE	Fathers of SGA infants: 181 g lighter at birth compared to fathers of non-SGA infants.	Auckland, New Zealand and Adelaide, Australia
					SGA: birthweight <10th centile	
[35]	Food availability during SGP	F_1_	320 individuals born in the year 1890, 1905 and 1920.	Överkalix cohort	Sons: protected from cardiovascular death if their father experienced poor availability of food during his SGP (odds ratio (OR): 0.42).	Sweden
[54]	BMI (pre-conceptional)	F_1_	11,784 children aged 7–18 years and their parents		Overweight in father: increased the risk of developing MS in children by 2.17 times.	China
					Overweight in father: positively correlated with risk of developing MS, obesity and low HDL cholesterol in both boys and girls.	
[39]	BMI (pre-conceptional)	F_1_	429 offspring and their parents from year 2017 to 2019.		Paternal BMI > 25: positively associated with offspring birthweight.	USA
					Paternal BMI: associated with DNA methylation status in blood of offspring at birth, age 3 and 7 years.	
[40]	BMI (pre-conceptional)	F_1_	33,448 pregnant women, including partners and infants.	JECS	Paternal BMI: positively correlated with the OR of LGA male infants (*p* = 0.01).	Japan
					Paternal BMI: weakly associated with the OR of LGA female infants (*p* = 0.04).	
[42]	BMI (pre-conceptional)	F_1_	2220 newborns (1155 boys and 1065 girls).	CBC	Paternal BMI had mild but significant effect (*p* < 0.05) on offspring BMI z score at the age of 2 years.	China
[61]	BMI (pre-conceptional)	F_1_	132,331 children.	MoBa and DNBC	In both cohort, paternal obesity was associated with increased risk of developing childhood type 1 diabetes.	Norway, Denmark
[43]	BMI (pre-conceptional)	F_1_	1494 parent-offspring pairs. Offspring were followed up at the age of 5, 14, and 21 years.		Paternal BMI *z*-score: positively associated with offspring BMI z-score from the age of 5–21 years. This association became stronger as offspring aged.	Australia
[62]	BMI (pre-conceptional)	F_1_	5327–5377 parents-offspring pairs from ALSPAC and NFBC86.	ALSPAC, NFBC66 and NFBC86.	In each cohort, paternal BMI was strongly positively associated with offspring VLDL cholesterol, VLDL triglycerides and negatively associated with offspring HDL, HDL_2_, and HDL_3_ cholesterol.	UK, Finland
			4841–4874 mother-offspring pairs from NFBC66.			
			Offspring blood was collected at the age of 16, 17, and 31 years.			
[56]	BMI (pre-conceptional)	F_1_	21 years old 2229 children and their parents.		For each unit increase in paternal BMI, the BMI and WC of offspring at the age of 21 years were increased by 0.33 kg/m^2^ and 0.76 cm respectively.	Australia
[41]	BMI (pre-conceptional)	F_1_	30,566 parents-offspring pairs. Offspring weight and BMI was collected at birth, 5 months, 1 year, and 7 years of age.	DNBC	At every time point, paternal BMI *z*-score was associated with offspring weight and BMI *z*-score.	Denmark
[52]	BMI (pre-conceptional)	F_1_	580 children (339 boys and 241 girls, mean age 9.6 years)		Paternal BMI was associated with elevated offspring BMI (*β* = 0.161, *p* < 0.001), WC (*β* = 0.404, *p* < 0.001), triglycerides (*β* = 0.017, *p* < 0.05), MRS (*β* = 0.084, *p* < 0.05), and CRF (*β* = −0.174, *p* < 0.001).	China
[65]	BMI (pre-conceptional)	F_1_	92 newborns and parents.	NEST	Increased paternal BMI: associated with hypomethylation at DMRs of MEST (*β* = −2.57; *p* < 0.01), PEG3 (*β* = −1.71; *p* < 0.01) and NNAT (*β* = −3.59; *p* < 0.05) in umbilical cord blood leukocytes of offspring.	USA
[64]	BMI (pre-conceptional)	F_1_	79 newborns and parents.	NEST	Increased paternal BMI: associated with hypomethylation at DMRs of *IGF-2* (*β* = −5.28, *p* < 0.01) in umbilical cord blood leukocytes of offspring.	USA
[51]	BMI (pre-conceptional)	F_1_	4871 parents-offspring pairs. Offspring mean age 6 years (ranging from 5.6 to 8 years).		Paternal BMI: positively associated with offspring BMI, AFM, SBP, insulin level, and negatively associated with HDL cholesterol levels (*p* < 0.05).	Rotterdam, The Netherlands
[50]	Adiposity (SS: sum of skinfolds) (pre-conceptional)	F_1_	504 children (at 9.5 years of age) and their parents.		Paternal SS: associated with increased offspring BMI, SS, fat percentage, WC, fasting insulin and IR.	Mysore, India
					For each unit SD increase in paternal BMI, the SD of offspring adiposity at the age of 9.5 years increased by 25%.	
[63]	BMI (pre-conceptional)	F_1_	899 parents-newborn pairs (492 newborn boys and 407 newborn girls).	Guangzhou Birth Cohort Study	Paternal BMI: associated with birth parameters and cortisol level of male but not female offspring.	Guangzhou, China
[57]	BMI (pre-conceptional)	F_1_	All people born in England, Wales and Scotland in March 1958.	1958 British Birth Cohort Study	For each unit increase in paternal BMI, offspring BMI at the age of 45 years was increased by 0.24–0.35 unit.	UK
[55]	BMI (pre-conceptional)	F_1_	16 years old 2325 boys, 2463 girls and their parents.		Paternal BMI: positively strongly correlated with developing overweight/obesity in children at the age of 16 years (father-son OR 3.17, 95% CI 1.70, 5.92; father-daughter OR 5.58, 95% CI 3.09, 10.07).	Finland
[44]	BMI (pre-conceptional)	F_1_	9346 total participants including parents. Offspring at 11 and 44–45 years of age.	1958 British Birth Cohort Study	Paternal BMI: positively correlated with offspring BMI in both childhood (11 years of age) and mid-adulthood (44–45 years of age).	UK
					For each unit increase in paternal BMI, the BMI of offspring at the age of 44–45 years increased by 0.21–0.29 unit.	
[45]	BMI (pre-conceptional)	F_1_	1483 adolescents (11 years old), 1156 mothers, and 1016 fathers		Paternal overweight and WC: strongly associated with overweight and WC of male offspring at the age of 11 years (*p* < 0.001 for both paternal factors).	Norway
[46]	BMI (pre-conceptional)	F_1_	741 boys and 689 girls. Children were followed up at the age of 1, 3, 6, and 8 years.	Raine cohort	Paternal overweight and obesity increased risk of overweight including obesity in children at the age of 8 years.	Australia
[53]	BMI (pre-conceptional)	F_1_	940 children (9.5 ± 0.4 years) and 873 adolescents (15.5 ± 0.5 years) and their parents.		Paternal BMI: positively associated with offspring BMI, WC and skin fold thickness (*p* < 0.001 for all measurements).	Estonia and Sweden
[47]	BMI (pre-conceptional)	F_1_	5–7-year-old 2631 children and their parents.	KOPS	Paternal overweight: independent risk factor in developing overweight and obesity in children of 5–7 years old.	Germany
[48]	BMI (pre-conceptional)	F1	9–18-year-old children from 219 families.		Paternal BMI: consistently positively associated with BMI of both male (*p* = 0.03) and female (*p* = 0.024) offspring over the period of 9 years.	Australia
					Male and female offspring had four-fold increased risk of being obese at age 18 if their father was obese.	
[58]	BMI (pre-conceptional)	F_1_	6540 men and 6207 women.	1958 British birth cohort	Paternal BMI: positively correlated with offspring BMI. Adult offspring from overweight or obese fathers were at high risk of being obese.	UK
[49]	BMI (pre-conceptional)	F_1_	676 boys, 687 girls, and their parents.		Paternal BMI: strongly associated with increased risk of childhood obesity in both sexes.	Italy
[60]	Metabolic Syndrome (MS) (pre-conceptional)	F_1_	785,809 births to healthy mothers and their male partners		Paternal MS: had an impact to increase odds of having preterm birth by 19% (95% CI 1.11–1.28), LBW by 23% (95% CI 1.01–1.51), and NICU stay by 28% (95% CI 1.08–1.52).	USA
[37]	Food availability during SGP	F_2_	303 individuals and their 1818 parents and grandparents from the year 1890, 1905, and 1920.	Överkalix cohort	Grandson’s mortality risk was associated with paternal grandfather’s food availability during his SGP.	Sweden
[35]	Food availability during SGP	F_2_	320 individuals born in the year 1890, 1905, and 1920.	Överkalix cohort	Diabetes related mortality risk for grandchild was increased if their grandfather experienced overnutrition during his SGP.	Sweden
[36]	Food availability during SGP	F_2_	94 individuals born in the year 1905.	Överkalix cohort	Grandchild’s longevity was shortened if the paternal grandfather experienced overnutrition during his SGP.	Sweden

*↑* Increased, *ALSPAC* Avon Longitudinal Study of Parents and Children, *AFM* abdominal fat mass, *BMI* body mass index, *CBC* Chinese Birth Cohort, *CI* confidence interval, *CRF* cardiorespiratory fitness, *DFBC* Dutch Famine Birth Cohort, *DMRs* differentially methylated regions, *DNBC* Danish National Birth Cohort, *HDL* high density lipoprotein, *IGF-2* insulin-like growth factor-2, *IR* insulin resistance, *JECS* Japan Environment and Children’s Study, *KOPS* Kiel Obesity Prevention Study, *LBW* low birthweight, *LGA* large for gestational age, *MEST* mesoderm specific transcript, *MoBa* Norwegian Mother and Child Cohort Study, *MS* metabolic syndrome, *NEST* Newborn Epigenetics Study, *NICU* neonatal intensive care unit, *NNAT* neuronatin, *MRS* metabolic risk score, *NFBC66* Northern Finland Birth Cohort 1966 study, *NFBC86* Northern Finland Birth Cohort 1986 study, *OR* odd ratio, *PEG3* paternally expressed gene-3, *SBP* systolic blood pressure, *SCOPE* screening for pregnancy endpoints, *SD* standard deviation, *SGA* small for gestational age, *SGP* slow growth period, *SS* sum of skinfolds, *VLDL* very low density lipoprotein, *WC* waist circumference.

**Table 2:** Relevant rodent studies showing paternal programming of offspring and grand-offspring metabolic health.

Reference	Animal model	Founder diet and duration	Affected generation	Offspring outcomes
[83]	Wistar rats	Control (10% fat, 20% protein, and 70% carbohydrate) and HFD (45% fat, 20% protein, and 35% carbohydrate) for 3 and 9 months respectively. HFD feeding started since lactation in corresponding dams	F_1_	HFD fed F_1_ males and females (PND-50 to PND-120): ↑ bodyweight.
				HFD fed F_1_ males (at PND-120): ↑ circulating leptin.
				HFD-induced glucose intolerance in offspring of both sexes were not affected by paternal obesity.
[78]	Sprague–Dawley (SD) rats	Control (Net energy 11 kJ/g, 13% fat, 22% protein, and 65% carbohydrate) or HFD (Net energy 20 kJ/g, 43% fat, 17% protein, and 40% carbohydrate) for 13–14 weeks	F_1_	F_1_ males: altered growth hormone, IGF-1 production, ↓ adipogenesis marker in fat pads and ↑ lipogenic genes in muscle.
[77]	SD rats	Control (Net energy 11 kJ/g, 12% fat, 21% protein, 65% carbohydrate) or HFD (Net energy 20 kJ/g, 43% fat, 17% protein, 40% carbohydrate) for 13–14 weeks	F_1_	F_1_ males: ↓ bodyweight, ↑ triglyceride content, tubular changes in kidney.
[84]	SD rats	Control (10% energy as fat) or HFD (45% energy as fat) for 16 weeks	F_1_ + F_2_	F_1_ females: ↑ bodyweight.
				F_2_ males from F_1_ females born to HFD fed founders: ↑ adiposity and plasma leptin.
				F_2_ males from F_1_ males born to HFD fed founders: no metabolic changes.
				F_2_ females from both parental lineage (F_1_) sired by HFD fed founders: no change in bodyweight, adiposity or size of organ.
[82]	Institute of Cancer Research (ICR) mouse	Control (Energy percentage: 12.8% fat, 25.6% protein and 61.6% carbohydrate) or HFD (Energy percentage: 62.0% fat from lard, 18.0% protein, and 20.0% carbohydrate) for 6 weeks	F_1_	F_1_ males and females: ↑ bodyweight, fat mass, impaired metabolic traits through epigenetic modification of adipocytokine and leptin gene.
[89]	A^vy^ mice derived from isogenic C57BL/6 mice	Control (5% w/w fat) or HFD (22% w/w fat) for 9 weeks	F_1_ + F_2_ + F_3_	HFD fed F_1_ males: defective glucose and lipid metabolism.
				The induced but latent metabolic traits in F_1_ males from obese mice father transmitted to F_2_ and F_3_ males in the absence of dietary challenge.
[81]	C57BL/6 mice	Control (Net energy: 16.5 kJ/g, 4% carbohydrates, 19% protein, and 17% fat) or HFD (Net energy: 20.7 kJ/g, 32% carbohydrates, 19% protein and 49% fat) for 8 weeks	F_1_	F_1_ males and females: impaired glucose metabolism and liver steatosis.
				HFD fed F_1_ males and females: amplified paternal programming.
[87]	C57BL/6 mice	Control (10% kcal energy as fat) or HFD (60% kcal energy as fat) for 10 weeks	F_1_	F_1_ males: altered expression of genes associated with oxidative stress and lipid metabolism.
[85]	C57BL/6 mice	Control (Net energy: 16.1 KJ/g, 14% protein, 21% fat) or HFD (Net energy: 19.4 KJ/g, 17% protein, 40% fat) for 12 weeks	F_1_	F_1_ males: ↑ increased adipose depots, serum leptin levels and ↓ glucose tolerance.
				HFD fed F_1_ male: amplified paternal programming.
[88]	SD rats	Control (energy content not disclosed) or HFD (42–45% energy as fat) for 12 weeks	F_1_ + F_2_	F_1_ and F_2_ pups: ↓ bodyweight.
				F_1_ female pups: ↓ reduced pancreatic beta-cell mass.
				F_1_ and F_2_ females: ↓ GT.
				F_2_ females: ↓ insulin level during GTT.
				F_2_ males: ↑ insulin level during GTT.
				HFD fed F_1_ and F_2_ females: ↑ resistance to HFD-induced weight gain.
				HFD fed F_2_ females: further impairments in GT.
				F_1_ and F_2_ males when exposed to HFD: did not show major phenotypic differences.
[86]	C57BL/6 mice	Control (Net energy: 2.7 kcal/g, 59.9% carbohydrate, 16.1% protein, 3.1% fat) or western diet (HFD + HSD) (Net energy: 4.1 kcal/g, 46.1% carbohydrate, 15.3% protein, 17.9% fat) for 4 months	F_1_	F_1_ males and females: ↑ bodyweight, ↓ GT and IS.
[76]	SD rats	Control (Net energy: 11 kJ/g, 12% fat, 21% protein, 65% carbohydrate) or HFD (Net energy: 20 kJ/g, 43% fat, 17% protein, 40% carbohydrate) for 11 weeks	F_1_	F_1_ females: differentially expressed gene related to ageing and chronic degenerative disorders in RpWAT and pancreatic islets.
[80]	C57BL/6 mice	Control (Net energy: 16.1 KJ/g, 14% protein, 21% fat) or HFD (Net energy: 19.4 KJ/g, 17% protein, 40% fat) for 10 weeks	F_1_ + F_2_	F_1_ males and females: ↓ GT and IS.
				F_1_ males and females: ↑ adiposity in sex specific way (predominantly in F_1_ female).
				F_2_ females from F_1_ females born to HFD fed founders: ↑ IR
				F_2_ males from F_1_ females born to HFD fed founders: ↑ bodyweight, ↓ GT and IS.
				F_2_ females from F_1_ males born to HFD fed founders: ↑ adiposity and IR
				F_2_ males from F_1_ males born to HFD fed founders: no metabolic changes.
[75]	SD rats	Control (Net energy: 11 kJ/g, 12% fat, 21% protein, 65% carbohydrate) or HFD (Net energy: 20 kJ/g, 43% fat, 17% protein, 40% carbohydrate) for 11 weeks	F_1_	F_1_ females: early onset of impaired insulin secretion and GT that worsened with time.
				F_1_ females: pancreatic beta-cell dysfunction and altered expression of 642 pancreatic islet genes.

N.B: The effects of paternal obesity on subsequent generations are reported in offspring outcomes.

*↑* Increased, *↓* Decreased, *HFD* high fat diet, *PND* postnatal day, *IGF-1* insulin-like growth factor-1, *GT* glucose tolerance, *GTT* glucose tolerance test, *IR* insulin resistance, *IS* insulin sensitivity.

**Table 3:** Relevant rodent studies showing paternal programming of offspring and grand-offspring reproductive health.

Reference	Animal model	Founder diet and duration	Affected generation	Offspring outcomes
[79]	C57BL/6 mice	Control (7.2% fat, 20.5% protein, and 61.6% carbohydrate) and HFD (36% fat, 20.5% protein, and 35.7% carbohydrate) for 99 days.	F_1_ + F_2_	F_1_ and F_2_ males: defective sperm morphology, altered testicular metabolites associated with insulin resistance, oxidative stress and defective sperm quality (count, viability, motility and morphology). F_2_ males: ↓ sperm counts.
[92]	Wistar rats	Control (Net energy: 3.86 Kcal/kg, 9% minerals, 22% protein, 5% ethereal extract, 7% fiber and 57% nitrogen-free extract) and high fat high sugar diet (HFHS) (Net energy: 4.77 Kcal/kg, 6.2% minerals, 23.7% protein, 23.9% ethereal extract, 4.5% fiber and 41.7% nitrogen-free extract) for 65 days.	F_1_ + F_2_	F_1_ males: ↓ TDA, ↓MGA, ↓MGB, ↑sperm count, ↓ VDT, ↑VDI, ↑VIT, ↓HE, ↑LYM, ↑VLEY, ↑VLYM, ↑VMAT.
				F_2_ males: ↑ MGA, ↓MGB, ↑MAT, ↑net testis weight, ↑ VPW.
[83]	Wistar rats	Control (10% fat, 20% protein, and 70% carbohydrate) and HFD (45% fat, 20% protein, and 35% carbohydrate) for 3 and 9 months respectively. HFD feeding started since lactation in corresponding dams.	F_1_	HFD fed F_1_ males and females: negatively affected LH responses to KP-10 predominantly in F_1_ males.
				HFD-induced hypogonadism in F_1_ males were amplified by paternal obesity.
[91]	C57BL/6 mice	Control (6% fat content) and HFD (22% fat content) for 12 weeks.	F_1_	F_1_ females: produced embryo with delayed development, had blastocysts with impaired quality.
				F_1_ females: ↑ expression of glucose transporter genes in ovary, ↑ GLUT4 gene expression in cumulus cells and ↑ lipid droplet content in cumulus oocyte complexes.
[85]	C57BL/6 mice	Control (Net energy: 16.1 KJ/g, 14% protein, 21% fat) or HFD (Net energy: 19.4 KJ/g, 17% protein, 40% fat) for 12 weeks.	F_1_	F_1_ males: ↓ sperm motility, ↓ sperm-oocyte binding capacity and ↑ sperm ROS level.
				HFD fed F_1_ males: amplified paternal programming.
[90]	C57BL/6 mice	Control (Energy percentage: 6% fat, 19% protein and 64.7% carbohydrate) and HFD (Energy percentage: 22% fat, 0.15% cholesterol, 19% protein and 49.5% carbohydrate) for 10 weeks.	F_1_ + F_2_	F_1_ males: ↓ sperm motility, fertilization capacity, ↑ sperm ROS and DNA damage.
				F_1_ females: ↓ meiotic competence of oocytes, ↑ mitochondrial membrane potential in all regions of oocytes (outer +17.5%; middle +62.4%; inner +57.1%).
				F_2_ males from F_1_ males sired by F_0_ obese founder: ↓ sperm motility, ↑ sperm ROS level.
		Sperm ROS production and oxidative DNA damage was mainly focused in mice founder (F0 males)		
				F_2_ females from F_1_ males sired by F_0_ obese founder: oocytes with increased oxidative stress and ↑ mitochondrial membrane potential in middle and inner part of oocyte.
				F_2_ males from F_1_ females sired by F_0_ obese founder: ↓ sperm testosterone level, ↓ sperm motility, ↑ sperm ROS level.
				F_2_ females from F_1_ females sired by F_0_ obese founder: ↓ ROS level in oocytes.

N.B: The effects of paternal obesity on subsequent generations are reported in offspring outcomes.

*↑* increased, *↓* decreased, *HFD* hight-fat diet, *HFHS* high fat high sugar diet, *LH* Luteinizing hormone, *KP-10* Kisspeptin-10, *GLUT-4* glucose transporter-4, *ROS* reactive oxygen species, *TDA* testicular descent (days) phase A, *MGA* morphology of the penis glans-phase B (days), *MGB* morphology of the penis glans-phase B (days), *VDT* volumetric density of the tubular testicular compartment, *VDI* volumetric density of the intertubular testicular compartment, *VIT* volume of the intertubular testicular compartment (mL), *VPW* relative weight of the ventral prostate (%), *HW* height of the seminiferous epithelium (μm), *LYM* volumetric density of lymphatic space, *VLEY* volume of Leydig cells (μl), *VLYM* lymphatic space volume (μl), *VMAT* extracellular matrix volume (μl), *MAT* volumetric density of the extracellular matrix.

## Obesity compromises sperm

Mounting evidence across species suggests that obesity can have negative impacts on conventional sperm parameters (sperm count, concentration, motility, viability, and morphology) [11, 17–23]. In a recent mice study, it is demonstrated that high-fat-diet (HFD)-induced obesity during childhood can cause irreversible damage to sperm quality in later life [23]. It is also evident that obesity is associated with increased reactive oxygen species (ROS) production and DNA damage in sperm [11, 24–29]. Such increased sperm DNA damage has been linked with adverse consequences in pregnancy [30] and offspring health outcomes, discussed later in this review. Given the current state of the global obesity epidemic, the number of fathers with obesity planning to conceive is increasing [31]. Therefore, an increasing impact on offspring health in years to come can easily be postulated.

## Evidence on paternal programming of offspring health and disease

### Human studies

In humans, there is limited evidence showing a causal effect of paternal obesity on future generations. Most of the studies to date have reported associative data (i.e., retrospective or cross-sectional studies). Therefore in this section we briefly discuss the effects of paternal undernutrition, birthweight, body mass index (BMI) as well as overnutrition and metabolic syndrome (MS) to gain some insight into how paternal environmental factors could program offspring health. Table 1 summarizes 33 human studies showing paternal programming effects on offspring health outcomes (sequentially listed based on paternal factors from prenatal to pre-conception exposures in father). It is evident that the father’s prenatal exposure to undernutrition has been found to impact their offspring bodyweight. Veenendaal et al. reported that the adult offspring from prenatally undernourished fathers during the 1944–1945 famine in the Netherlands (Dutch Famine Birth Cohort Study) had increased bodyweight (+4.9 kg) and BMI (+1.6 kg/m^2^) [32]. An association was also observed between father and offspring birthweight. Two separate studies from the same group reported that fathers of large for gestational age (LGA) infants were 180 g heavier [33] and fathers of small for gestational age (SGA) infants were 181 g lighter [34] at birth compared to fathers of non-LGA and non-SGA infants respectively. It thus suggests a positive association of father’s birthweight with their offspring’s birthweight. However, prenatal nutritional exposure of father underpinning increased birthweight was not mentioned in those studies [33, 34].

It is concerning that paternal nutritional status before puberty can affect the health outcomes of their offspring as well as grand-offspring. In the Överkalix study by Kaati et al., nutritional restriction in fathers during their slow-growth-period (SGP between 9 and 12 years of age) protected their sons from CVD-related death. Interestingly, paternal grandfather’s overnutrition during their SGP increased the risk of diabetes-related death of their grand-offspring (Odds Ratio 4.1, *p* = 0.01) [35]. Work from the same group reported that a grandchild’s longevity can be shortened if the paternal grandfather experienced overnutrition during his SGP in comparison with the grandchild whose grandfather experienced undernutrition during SGP [36]. They also found that the transgenerational effect was sex specific, whereby paternal grandfather’s food supply was only linked to the mortality risk of grandsons, while paternal grandmother’s food supply was only associated with the granddaughters’ mortality [37].

Emerging evidence suggests that a high paternal pre-conception BMI is a potential risk factor in compromising embryo development and pregnancy health. In a study among couples undergoing assisted reproductive treatment in South Australia, increased paternal BMI was associated with decreased pregnancy rate, implantation rate, blastocyst development rate and live birth [38]. Furthermore, human studies highlighted a paternal contribution (BMI and/or waist circumference (WC)) to increased birthweight [39–41], increased risk of offspring developing obesity in infancy (0–3 years) [40–42], childhood (4–12 years) [41, 43–54], adolescence (13–19 years) [48, 53–55], early adulthood (20–30 years) [56] and late adulthood (40–50 years) [44, 57, 58].

Very recently, MS in fathers before conception has been reported to be associated with increased preeclampsia of mother during pregnancy [59, 60], preterm birth, low birthweight and NICU stay [60]. It is also concerning that not only offspring adiposity but also associated metabolic comorbidities in childhood [50–52, 54, 61], adolescence [54, 62] and adulthood [62] have been closely linked with paternal BMI. Interestingly, some sex specific impacts of paternal BMI on offspring health outcomes were observed. In a Chinese birth cohort study Chen et al reported that paternal BMI before conception was associated with fetal growth of male offspring but not female offspring [63].

Paternal obesity has been shown to be associated with altered epigenetic marks in offspring. Work by Soubry et al. reported a negative association between paternal obesity and DNA methylation in offspring suggesting that increased paternal BMI was associated with hypomethylation at the differentially methylated regions (DMRs) of the imprinted insulin-like growth factor-2 (IGF-2) [64], mesoderm specific transcript (MEST), paternally expressed gene-3 (PEG3), and neuronatin (NNAT) [65] genes in umbilical cord blood leukocytes of offspring. IGF-2 [66], MEST, PEG3 [67] and NNAT [68] play significant role in prenatal and postnatal growth regulation and dysregulation of any of these genes are associated with developing obesity. These studies indicate that paternal obesity can impair fetal growth which might relate to the increased risk of offspring developing obesity in childhood and adulthood as discussed previously.

However, unlike paternal nutritional status before puberty, the effects of paternal BMI and/or WC on the health outcomes of grandoffspring have not been well documented. Therefore, further human studies are warranted to investigate the potential impacts of paternal obesity during conception on the health outcomes of future generations.

Given these epidemiological and clinical findings, it is clear that the prenatal to pre-conception paternal environmental factors could be potential contributors in programming health outcomes of future generations. However, in human given the inter-generational time, it is very difficult to observe the effects of male obesity on several successive generations. It is also difficult to obtain specific tissue samples from the offspring to ascertain the effects of paternal obesity at the cellular and molecular level. Therefore, animal models are of particular interest in exploring the transmission of paternal effects to future generations.

### Animal studies

Most animal studies on paternal programming effects have been performed in rat and mouse models. Emerging evidence from animal studies suggested that obesity in fathers can negatively program embryo development as well as the metabolic (Table 2) and reproductive (Table 3) health outcomes of multiple generations.

#### Paternal programming of embryo development

Animal studies suggest that diet-induced paternal obesity is closely associated with impaired embryo development leading to associated complications. In this setting, Binder et al. revealed in mice that HFD-induced paternal obesity could delay fertilization of the oocyte, cell cycle progression during the second and third cleavage event of embryo development, reduce blastocyst outgrowth, fetal and placental weight, crown-rump length and thus impair fetal development [69, 70]. Furthermore, they found that paternal obesity can affect the placenta in a sex-specific manner as indicated by decreased expression of Ppara, Casp12 in only male placenta, while female placenta had increased global DNA methylation [71]. Reduced expression of Ppara, Casp12 is a clear indication of increased cellular damage [72] while increased DNA methylation could lead to fetal growth restriction, even fetal death [73]. Interestingly, sex-specific effects on the development of the embryo could also be observed before the pre-implantation period. For example, microarray analysis of blastocysts from obese mice fathers revealed that 49 differentially abundant transcripts were upregulated in male blastocysts, while in female, most of the differentially abundant transcripts were downregulated (47 down, 2 up) [74]. Given this evidence, it is becoming apparent that paternal obesity can negatively program embryos across various stages of developments.

#### Paternal programming of F_1_ metabolic health

It is evident that offspring metabolic health can potentially be programmed by paternal overnutrition as demonstrated across many rodent studies (Table 2).

The effect of diet-induced paternal obesity on offspring health outcomes was pioneered by our laboratory in 2010. We reported that female offspring of chronic HFD fed rat fathers had impaired glucose metabolism, pancreatic beta-cell dysfunction [75], ageing and chronic degenerative disorders related gene expression changes in retroperitoneal white adipose tissue (RpWAT) and pancreatic islets [76]. Two subsequent findings from our lab demonstrated that male offspring from obese fathers can also be affected as indicated by reduced bodyweight, impaired lipid deposition with tubular changes in kidney [77], altered growth hormone, insulin-like growth factor-1 (IGF-1) production, decreased adipogenesis marker in fat pads and upregulated lipogenic genes in muscle [78].

Mounting evidence from other rodent studies has also demonstrated adverse programming effects of diet-induced paternal obesity on metabolic health particularly bodyweight, adiposity and glucose metabolism of F_1_ offspring (Table 2). In rodents, feeding HFD (for 6–15 weeks) in fathers was found to increase weaning bodyweight in male offspring [79], increase bodyweight, impaire glucose metabolism in both male and female offspring [80–85]. Western style diet (WD, containing HFD plus high sucrose) induced paternal obesity has also been shown to increase bodyweight, impair glucose metabolism and insulin sensitivity in male and female mice offspring [86].

Increased risk of fat deposition and inflammation in liver has also been seen in offspring from obese fathers. Ornellas et al. reported that paternal HFD for 8 weeks in mice induced lipogenesis and liver steatosis in male and female offspring [81]. Further evidence was provided by Terashima et al. indicating dysregulated expression of lipogenesis genes in livers of male mice offspring from HFD fed fathers [87]. Evidence from animal models also support that paternal obesity can exert sex-specific metabolic effects on their offspring [80, 88] (see description in Table 2).

It is thus apparent that diet-induced paternal obesity can compromise the metabolic health of offspring. Interestingly, diet-induced obesity in fathers can also predispose their offspring to a latent metabolic syndrome which could be unmasked if the offspring are exposed to a post weaning dietary challenge. Cropley et al. reported that CD fed male mice offspring from obese fathers did not develop glucose intolerance, hyperinsulinemia, abnormalities in hepatic lipid metabolism until they were exposed to post weaning WD challenge compared to WD fed offspring sired by lean father, suggesting a latent predisposition of hepatic insulin resistance in male offspring by obese father [89].

#### Paternal programming of F_2_ metabolic health

Emerging evidence from rodent studies suggests that diet-induced obesity in fathers can also program the metabolic health of F_2_ and even F_3_ generation. For instance, newborn and adult F_2_ rat offspring from HFD fed grandfather had reduced bodyweight and glucose tolerance respectively, similar to F_1_ offspring [88]. Cropley et al. reported in mice that WD induced grandpaternal obesity predisposed their F_2_ males to mild hyperinsulinemia and slightly impaired glucose tolerance. They also showed that the programmed but latent metabolic phenotype (glucose intolerance and defective hepatic lipid metabolism) in control fed F_1_ males of obese mice father could be transmitted to WD fed F_2_ and F_3_ males suggesting that F_1_ males in the absence of the dietary challenge can transmit metabolic defects to their F_2_ and F_3_ progeny [89]. It is also evident that paternal obesity can alter metabolic phenotype in grand-offspring in a parental lineage (F_1_) and sex (F_2_) specific manner (see Table 2) [80, 84]. Additional evidence supporting sex specific transgenerational effects of paternal obesity in rats was reported by de Castro et al. where CD fed F_2_ female (but not F_2_ male) sired from obese grandfather had reduced insulin levels during a glucose tolerance test [88].

It is thus evident that the metabolic outcomes in the offspring and grandoffspring is affected by a number of factors including the diet consumed, exposure time before conception, species (and strain), level of metabolic defects in the parents and challenges faced by the offspring and grandoffspring. Clearly such findings strongly indicate an impact of the paternal pre-conceptional environment (obesity/overnutrition) on the metabolic health of subsequent generations, underlining the need to intervene.

#### Paternal programming of F_1_ and/or F_2_ reproductive health

Reproductive health (e.g., oxidative stress level and fertilization capacity of sperm and oocyte, embryo development, sex hormone regulation, metabolomic, histometric and volumetric analysis of reproductive organ) of subsequent generations can be perturbed like metabolic health by paternal obesity before conception as evidenced by several rodent studies (detailed in Table 3). In this context, Fullston and colleagues reported several remarkable outcomes in mice. For instance, paternal obesity has been shown to decrease sperm motility, sperm fertilization capacity, increase ROS level and sperm DNA damage in F_1_ male [85], both F_1_ and F_2_ male [90] and decrease oocyte fertilization capacity, increase ROS level in oocytes of female offspring up to the second generation [90]. In another study, Fullston et al., reported that F_1_ females born to HFD fed F_0_ male founders produced embryos with delayed development (especially during 2-cell and 8-cell stage), blastocysts with impaired quality (increased trophoblast cell number and decreased proportion of embryoblast). They also observed that such abnormalities in embryo and blastocysts development could be associated with molecular alterations in these offsprings’ ovaries and increased lipid accumulation in cumulus/oocyte complexes [91]. A recent study in Wistar rats reported that both F_1_ and F_2_ males born to high fat high sugar (HFHS) diet fed F_0_ males had early prepubertal development, altered volumetric density of testicular compartments, epididymis, seminal vesicle and seminiferous tubule [92]. Crisostomo et al. reported in C57BL6/J mice that both F_1_ and F_2_ males born to HFD fed F_0_ males had altered testicular metabolites associated with insulin resistance, oxidative stress and defective sperm quality (count, motility, viability and morphology) [79].

Like metabolic programming, reproductive health of future generations has also been found to be affected by paternal obesity in a sex-specific way. An evidence supporting sex-specific reproductive complications has been shown in a recent rat study where paternal obesity strongly perturbed hypothalamic pituitary gonadal axis of HFD fed male but not female offspring [83]. Overall, it is thus apparent that the paternal obesity at conception can compromise sperm and oocyte quality, perturb embryo development and increase oxidative stress in the sperm and oocyte of their offspring. Such induction of oxidative stress could lie behind the transgenerational effects of paternal programming, as further discussed in the next section.

### Mechanisms underlying obesity-induced paternal programming

As discussed earlier, it is now well documented from both human and animal studies that obesity can impair sperm quality in father. This impaired quality of sperm in father may result in compromised pregnancy health, impaired embryo development, fetal growth and increased risk of developing obesity and associated metabolic and reproductive complications in subsequent generations [38, 69, 70, 93]. Such predisposition may lead to a vicious cycle of obesity and associated comorbidities across several generations (depicted in Fig. 1 above). Sperm is the most critical element affected by the fathers health status that contributes to program offspring health. However, the underlying mechanism(s) responsible for obesity-induced paternal programming remains unclear. Several mechanisms underpinning obesity-induced paternal programming of offspring health have been proposed. One possible mechanism is molecular alteration of seminal composition which can perturb sperm integrity [94]. Emerging evidence from both human [95–98] and animal [99, 100] studies suggest that obesity has detrimental impact on seminal fluid composition. Another possible pathway is obesity-induced sperm epigenetic modification (microRNA, DNA or histones methylation, or acetylation) in father which might perturb transcription and translation of paternally derived genes during early embryogenesis [93]. A growing number of studies revealed the potential role of sperm epigenetic marks e.g., DNA methylation changes [88, 101, 102], histone modification [87], small non-coding RNAs [80, 86, 88, 103, 104], tRNA-derived small RNAs [104, 105], microRNAs, ribosomal RNA-derived small RNAs and long non-coding RNAs [104] in the context of paternal programming. Lastly, obesity-induced sperm oxidative DNA damage in fathers, leading to de novo mutations in embryos [106–108] is also being considered another possible mechanistic pathway. It is believed that epigenetic modification and oxidative DNA damage (measured as 8-OHdG, a ubiquitous and stable marker of oxidative DNA damage) in sperm are closely associated with each other [109, 110]. However, the likely role of paternal sperm DNA damage on subsequent offspring health has not been clearly documented.

### Sperm DNA damage and programming of offspring health

Evidence from human studies supporting a detrimental impact of sperm DNA damage in programming offspring health is mostly limited to fertilization, pregnancy rates, embryo developments and live birth rates [111–115]. Studies including couples undergoing assisted reproductive technology (ART) have found a clear association between sperm DNA fragmentation index (DFI, a marker of sperm DNA damage) and ART success. DFI is negatively correlated to embryo development (from day 2 to day 5), implantation rates and post-implantation embryo development [112, 113]. Increased sperm DFI has also been linked to increased pregnancy loss among couples seeking ART [97]. It should be noted that sperm DNA damage is most likely induced by oxidative stress, rather than defective apoptosis [116–118].

Unlike human studies, animal studies have been able to demonstrate a potential role of sperm DNA damage on offspring body composition, metabolic health, and mortality rates in addition to the effects on embryo and pregnancy outcomes. In mice, increased ROS in sperm (a measure of sperm DNA damage) induced by in-vitro H_2_O_2_ treatment has been linked with increased adiposity and impaired glucose metabolism in adult female offspring and increased adiposity in male offspring at 4 weeks of age [106]. In another mice study from the same lab, paternal diet restriction (70% ad libitum intake of control diet) for 17 weeks was found to increase sperm oxidative DNA lesions in father. This sperm DNA damage (as measured by 8-OhdG positive sperm) was negatively correlated with bodyweight of male offspring from PND5 to PND21 (*p* < 0.001) indicating a postnatal growth restriction induced by increased sperm DNA damage in father [119]. It has been shown that prenatal and early postnatal growth restriction is associated with increased adiposity in later life [120]. Hence the likely role of sperm DNA damage on developing obesity in adult offspring could easily be speculated. However, literature supporting the effect of sperm DNA damage is very limited in the setting of an obesity model. Very few studies have been reported where the likely role of obesity-induced sperm oxidative DNA damage on paternal programming of embryo development [11] and reproductive health of subsequent generations [90] (see detailed in Table 3) were investigated in mice. McPherson et al revealed that the increased sperm DNA damage (8-OHdG level) in father was linked to elevated oxidative DNA damage in paternal pronuclei, reduced percentage of embryonic 2-cell cleavage rates, increased fetal weight, decreased placental weight and increased fetal placental weight ratio [11]. However, no studies have particularly focused on the impact of obesity-induced sperm DNA damage on metabolic health of offspring.

Given the effect of obesity-induced sperm DNA damage on fetal outcomes and offspring reproductive health, further paternal programming of offspring metabolic and reproductive health could easily be speculated. Hence sperm DNA damage could be considered as potential mechanistic pathways underpinning obesity-induced paternal programming which requires further attention.

## Interventions to minimize paternal obesity-induced adverse programming of offspring health

It is now clear that paternal obesity can have adverse effects on offspring health through developmental programming, which may have significant contributions to the current epidemic of obesity and related health complications. Since obesity is a major contributor to the compromised sperm, interventions lowering obesity and obesity-induced sperm damage, sperm epigenetic modifications or promoting healthy sperm and seminal plasma may help reduce this cycle of POHaD (depicted in Fig. 1). The duration of spermatogenesis in human is 74 days, while it is 54 and 35 days in rat and mice respectively [121], any potential intervention should be implemeted to cover this sensitive time window before conception to rescue sperm from previous damage.

**Fig. 2:**
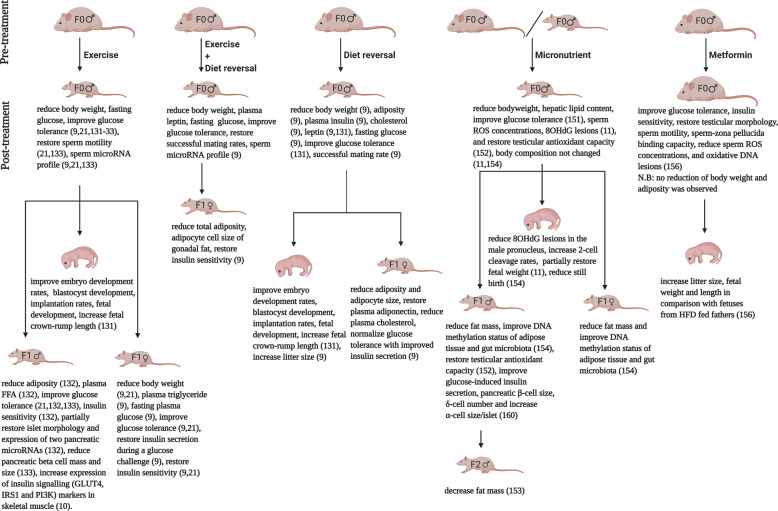
Schematic demonstration of paternal interventions to target obesity-induced paternal programming (created with BioRender.com). To date, interventions namely exercise, diet reversal, micronutrient and metformin in fathers have been implemented to combat paternal obesity-induced programming of offspring health outcomes.

Several strategies can be applied to improve paternal obesity, most of which involve weight loss commonly via dietary regulation, exercise and lifestyle changes [122]. In a recent study, a 12-week weight loss intervention consisting of healthy diet and daily exercise among 121 obese individuals has been shown to reduce BMI by 8.2% which resulted in reduced sperm DNA damage (measured by DFI) by 13.4%, indicating a positive association of weight loss with sperm DNA damage reduction [27]. A similar intervention in another study among 43 obese adults for 14 weeks was found to reduce BMI by 15%, which was associated with significant increased total sperm count, semen volume, normalized sperm morphology, increased serum testosterone, sex hormone-binding globulin (SHBG), and anti-Müllerian hormone [20]. In the case of morbid obesity, individuals are increasingly undergoing bariatric surgery to promote immediate weight loss and rectification of comorbidities [123]. In a recent 12 year follow up study among 1156 patients, bariatric surgery was shown to be very effective in reducing bodyweight and obesity-associated comorbidities (e.g., T2DM, hypertension, dyslipidaemia) [124]. Notably, massive weight loss from bariatric surgery was associated with improved sex hormone, sperm motility, count, viability and decreased seminal interleukin-8 levels (a marker of male genital tract inflammation) and sperm DFI [125]. Furthermore, in a recent systematic review and meta-analysis, sustained weight loss post-bariatric surgery has been reported to increase circulating testosterone, LH, FSH, SHBG, erectile function and thus improve sex regulating hormone in obese males [126]. There is also evidence in the literature that, many anti-obesity strategies have been linked to improve sperm epigenetic profile in human [127–130]. However, in humans, positive effects of weight loss by any intervention are limited to obese father; no intervention study has been conducted to examine the effects of paternal weight loss on offspring health. For practical reasons, such questions are more readily implemented in animals, and rodent studies have revealed the potential effects of various interventions designed to interrupt paternal obesity-induced programming of offspring health. To date, five types of intervention in obese rodents, namely exercise, shift to healthy diet/low-calorie diet, combined exercise and dietary modifications, micronutrient supplementation and drug treatment have been performed, with the potential effects followed in offspring (summarized in Fig. 2).

### Exercise intervention

Several rodent studies have applied pre-conceptional exercise in fathers to interrupt the programming effects of paternal obesity on different developmental stages of offspring. In this context, McPherson et al. reported that an exercise (swimming) intervention for 9 weeks before conception reduced bodyweight, total adiposity, improved glucose metabolism [9, 131, 132] and normalized sperm microRNA mir-503, increased abundance of mir-542-3p, and mir465b-5p [9] in HFD-induced obese mice founders. Notably this exercise intervention in obese father has been linked to improved embryo development rates, increased fetal weight (+16.4%) and crown-rump length (+6.3%) [131], reduced bodyweight (−3.5%), fasting plasma glucose (−8.8%), reduced plasma FFA, triglyceride, improved glucose tolerance and restored insulin sensitivity in female offspring. They suggested that normalization of X-linked microRNA mir-503 (regulates cell cycle after fertilization), increased abundance of mir-542-3p (regulates apoptosis), and mir465b-5p (regulates female fertility) in the sperm of obese fathers undergoing exercise intervention could mediate positive effects on female offspring [9]. In another study from the same lab, exercise intervention (swimming) for 9 weeks in obese mice founders reduced adiposity by 10.7%, circulating FFA and cholesterol level by 7% and 3.7% respectively, improved glucose metabolism and partially restored the function and morphology of pancreas in male offspring [132].

Further evidence supporting beneficial effects of exercise in obese fathers has been reported in several recent rodent studies. For instance, 3 weeks of exercise intervention (running wheels) was found to reduce bodyweight, fasting blood glucose and insulin, improve glucose tolerance, insulin sensitivity, restore sperm motility and sperm RNA profile in HFD fed mice founders [21, 133]. This exercise intervention resulted in reduced bodyweight in adult female offspring [21], improved glucose metabolism in both adult male and female offspring [21, 133] and restored pancreas morphology in adult male offspring [133]. Interestingly, in comparison with the studies by McPherson and her colleagues where obese founders were exercised for 9 weeks [9, 131, 132], it was observed that a 3-week running wheel intervention [21, 133] was more beneficial in the context of metabolic outcomes in obese fathers. There might be two possible reasons for this difference. First, in those studies by McPherson and colleagues, mice were fed HFD for 9 weeks before the 9-week intervention started, resulting in a more pronounced weight gain in HFD founders than in studies by Zheng et al. [133] and Stanford et al. [21]. Mice in these two latter studies were fed HFD along with access to running wheels for 3 weeks which prevented weight gain and development of obesity in the founders fed a HFD. Therefore, this 3-week intervention protocol had the chance to counteract HFD-induced damage as it was occurring.

Exercise intervention in obese fathers has also been found to exert a range of beneficial effects in the offspring. Krout et al. reported that paternal exercise (running wheels for 3 months) protected their male offspring from paternal HFD-induced insulin resistance by increasing expression of insulin signaling genes GLUT4, IRS1, and PI3K in skeletal muscle, thus decreasing the risk of developing T2DM [10]. Interestingly, in a recent mouse study, 6 weeks swimming in control diet fed fathers protected their offspring from HFD-induced liver steatosis, suggesting that an exercise intervention, even in fathers eating a healthy diet, can moderate offspring health outcomes [134]. Taken together it is apparent from rodent studies that exercise intervention in obese males may improve embryo development, and metabolic health of both male and female offspring.

### Diet reversal intervention

To date there has been only two studies investigating the benefits of shifting from an unhealthy diet to control diet before conception in male rodents. It was reported that when male mice were switched to control diet for 9 weeks after 9 weeks of HFD feeding, their bodyweight and total adiposity were reduced, net lean mass and percentage of lean mass were increased [9, 131], glucose tolerance was improved, circulating insulin, cholesterol and leptin level were reduced and sperm microRNAs (mir-503, mir465b-5p, and mir-542-3p) were normalized [9]. The resulting blastocysts from the diet switched father had improved development as evident by increased number of developing embryo, blastocyst total cell number, which led to normalized fetal weight, crown-rump length [131] and increased litter size [9]. Moreover, female offspring sired by these fathers had reduced bodyweight until 4 weeks of age, and normalized adipocyte cell size at 10 and 18 weeks of age, restored circulating adiponectin at 10 weeks of age, reduced circulating cholesterol at 18 weeks of age and normal glucose tolerance at 8 and 16 weeks of age. These improved metabolic outcomes in female offspring were argued to be the result of normalized X-linked sperm microRNAs in diet reversed obese fathers [9].

These beneficial outcomes demonstrate that reversal to a healthy diet, around the time of conception, can generate a positive environment for metabolic health and developing sperm in obese males and thus may minimize programming effect in future generations. However, the duration of the diet should be adequate to be able to rescue sperm and semen from previous impacts.

### Combined exercise and diet reversal intervention

To date only one study in rodents has been reported combining both exercise and diet reversal. As expected, combined exercise and diet intervention for 9 weeks was shown to improve pre-conception metabolic outcomes (reduce bodyweight, adiposity, increase glucose tolerance, reduce circulatory glucose, leptin, FFA, and inflammation level), normalize X-linked sperm microRNAs mir-503, mir-542-3p, and mir465b-5p in obese mice father. The combined intervention was also able to restore successful mating rate to a greater extent than exercise or control diet intervention alone in obese fathers. F_1_ female offspring from intervened obese fathers had reduced total adiposity (−11.8%) at 10 weeks of age, normalized glucose tolerance at 8 weeks of age, restored insulin sensitivity at 9 weeks of age. Like exercise or diet intervention alone, normalization of X-linked sperm microRNAs mir-503, mir-542-3p, and mir465b-5p in fathers by combining exercise and diet reversal may mediate the positive effects on offspring, as suggested by the authors [9].

### Micronutrient intervention

Micronutrients are vitamins and minerals required in small amounts in the diet and are essential for normal cellular and molecular functions [135]. Emerging evidence suggests that micronutrient deficiencies are highly prevalent in overweight/obese people compared to normal adults [136–142]. Moreover, micronutrient deficiency has been linked with decreased sperm quality [143]. On the other hand, mounting evidence suggests the beneficial effects of micronutrient supplementation to improve sperm conventional parameters (e.g., sperm counts, motility, morphology etc), reduce ROS production, DNA damage, increase antioxidant capacity, improve DNA integrity of sperm in sub fertile, and infertile patients [144–149], infertile rodents [150], undernourished rodents [119] and obese rodents [11]. However, evidence supporting micronutrient interventions to attenuate paternal obesity-induced programming of offspring health is very limited. Very recently, we have shown that micronutrient supplementation (containing folate, vitamin B6, choline, betaine, and zinc) for 27 weeks was able to reduce bodyweight, improve glucose metabolism and reduce hepatic lipid accumulation in HFD fed rat founders [151]. Most notably, in the same cohort, we have also shown that purtured testicular antioxidant capacity in father and their male offspring induced by paternal obesity was restored by the above mentioned supplementation in HFD fed father [152]. Moreover, male grand-offspring sired by supplemented HFD fed founders had reduced fat mass indicating potential beneficial effect of micronutrient supplementation in mitigating transgenerational effects of paternal obesity [153]. Furthermore, a decreased DNA damage in sperm was observed in our supplemented HFD fed founders compared to HFD fed founders (data not published yet). Therefore it could be interesting to see how sperm DNA damage in obese rat founders could relate with metabolic and/or reproductive complications in future generations and how this paternal programming could be mitigated by micronutrient supplementation.

In SD rats, micronutrient supplement containing folic acid (5.5 mg/kg diet), vitamin B_12_ (0.5 mg g/kg diet), betaine (5 g/kg diet), and choline (5.37 g/kg diet) for 9 weeks in high fat high sucrose diet (HFSD) fed father has been found to reduce stillbirth of offspring, reduce fat mass in both adult male and female offspring. In addition, father who fed supplemented HFSD had an impact to improve DNA methylation status of adipose tissue and gut microbiota in offspring of either sex. Interestingly, all these beneficial effects were observed without any change in body composition of supplemented HFSD fed founders compared to HFSD fed founders. However, supplementation appeared to improve insulin sensitivity of HFSD fed founders [154]. McPherson et al. reported that in mice, a 12-day micronutrient intervention (folic acid 1.5 mg/kg, zinc 61 mg/kg, vit C 700 mg/kg, vit E 78 mg/kg, lycopene 0.3 mg/kg, selenium 0.44 mg/kg and green tea extract 0.95 mg/kg) after feeding HFD for 10 weeks did not change bodyweight, adiposity, blood glucose and lipid profile but strikingly reduced sperm ROS concentrations and sperm oxidative DNA damage (8-OHdG) in supplemented HFD fed fathers, reduced the level of 8-OHdG in the male pronucleus, improved embryo development, and partially restored fetal weight [11]. This 12 days intervention was aimed to reduce ROS concentration in mature sperm during epidydimal transit where sperm spends around 9.5 days in mice [155]. However, intervention for 12 days in mice is not adequate to cover a full cycle of spermatogenesis which is 35 days [121]. In line with this, an intervention over 35 days was able to improve metabolic outcomes in obese fathers, as reported in the latest study by the same group [156].

Taurine (an essential amino acid) has beneficial impacts on male reproduction and metabolic health [157–159]. However very little is known about the effect of taurine supplement on the paternal programming of offspring metabolic health. In this context, Freitas and colleagues investigated how taurine supplementation in father could attenuate paternal programming of offspring health. They reported that in C57Bl/6 mice taurine supplementation (5%) for 4 months in HFD fed fathers didn’t change adiposity, glucose tolerance and insulin secretion in fathers but interestingly increased glucose-induced insulin secretion, normalized pancreatic β-cell size, increased α-cell size and δ-cell number in their adult male offspring. However, they didn’t investigate the effects of taurine supplementation on sperm of obese fathers [160].

Having seen the beneficial effects of micronutrient supplement, micronutrient intervention for a certain duration would be a novel approach to minimize paternal obesity-induced programming of offspring health outcomes. However, some care is needed to prevent negative effects from overdose thus further research is still warranted to examine whether dietary micronutrient supplementation in obese males can prevent a vicious cycle of paternal programming of disease in the progeny.

### Drug intervention

Apart from exercise, healthy diet/dietary modifications and micronutrient interventions, very recently metformin (a glucose lowering drug) for 6 weeks in HFD-induced obese fathers was found to improve glucose tolerance, insulin sensitivity as evident by reduced HOMA-IR level (−36.2%), restore testicular morphology, sperm motility (+157%), sperm-zona pellucida binding capacity (+33.1%), reduce sperm intracellular ROS concentrations, percentage of 8-OHdG positive sperm in obese founders, increase litter size (+22.7%), fetal weight (+5%) and fetal length (+2.1%) compared to fetuses from HFD fed fathers. Interestingly these beneficial effects in obese fathers and their fetuses were observed without any reduction of bodyweight and adiposity in the obese fathers [156]. Hence, this study indicates that promoting glucose tolerance independent of adiposity in fathers could be a new window to reduce damage in their sperm thus combat paternal obesity-induced programming of offspring health.

## Future studies

Emerging evidence suggests that perturbed sperm quality (increased sperm oxidative damage, altered sperm epigenetic profile, and seminal plasma composition) related to consumption of energy dense foods is linked to obesity-induced paternal programming of offspring health. However, such links are mostly derived from association studies. Therefore, there is an urgent need to conduct interventional studies investigating mechanism(s) to verify any causal links of offspring outcomes with increased sperm DNA damage, altered sperm epigenetic profile or altered seminal plasma composition of fathers.

To date, interventional studies in rodents (particularly exercise, healthy diet and micronutrient supplements) before conception in fathers have been mostly confined to target the effects of paternal obesity on embryo, fetal outcomes and metabolic health of the first generation. To the best of our knowledge, no previous studies have reported whether any intervention in fathers could ameliorate effects beyond the first generation, which is an important area for future investigation.

Also, as far we are aware, micronutrient intervention studies in rodents to ameliorate the effects of paternal obesity on future generation are mostly limited to using folate, zinc, betaine and choline intervention in fathers. It is thus necessary to further investigate interventional studies using other essential micronutrients such as vitamin A, vitamin B_6_, vitamin B_12_, vitamin C, vitamin D and vitamin E.

In addition, future studies should investigate whether glucose lowering drugs such as metformin can be advantageous to combat the effects of paternal obesity on subsequent generations. The optimal window for implementing any intervention to mitigate obesity-induced paternal programming of offspring health also further needs to be investigated.

The mechanistic and interventional studies related to obesity-induced paternal programming are largely confined to animal studies. Therefore, it is important to conduct human study frequently to determine the likely way of disease transmission from father to subsequent generations and its possible interventions.

## Conclusion

The present literature review strongly suggests that diet-induced obesity in fathers around the time of conception has substantial impacts to negatively program the health of multiple generations. It is thus necessary to increase the community awareness about the importance of a father’s health for better offspring health outcomes. This review also implies that sperm oxidative DNA damage in a father with obesity may have a critical role in the programming of offspring health.

Evidence from animal studies to date utilizing exercise, healthy diet, micronutrient supplements and metformin suggest that lifestyle modification, increasing the micronutrient content in energy dense food and improving metabolic health of a father around the time of conception could be ideal approaches to ameliorate obesity-induced paternal programming. The in-depth understanding of the mechanism(s) underpinning obesity-induced paternal programming and the benefits of interventions can help stop the vicious cycle of obesity and associated comorbidities across multiple generations.

## References

[1] The GBD. (2017). 2015 Obesity Collaborators. Health Effects of Overweight and Obesity in 195 Countries over 25 Years. N. Engl J Med.

[2] Kopelman PG (2000). Obesity as a medical problem. Nature..

[3] Nicolaidis S (2019). Environment and obesity. Metabolism..

[4] Fleming TP, Watkins AJ, Velazquez MA, Mathers JC, Prentice AM, Stephenson J (2018). Origins of lifetime health around the time of conception: causes and consequences. Lancet..

[5] Stephenson J, Heslehurst N, Hall J, Schoenaker D, Hutchinson J, Cade JE (2018). Before the beginning: nutrition and lifestyle in the preconception period and its importance for future health. Lancet..

[6] Portha B, Grandjean V, Movassat J (2019). Mother or Father: Who Is in the Front Line? Mechanisms Underlying the Non-Genomic Transmission of Obesity/Diabetes via the Maternal or the Paternal Line. Nutrients..

[7] Li J, Tsuprykov O, Yang X, Hocher B (2016). Paternal programming of offspring cardiometabolic diseases in later life. J Hypertens.

[8] Houfflyn S, Matthys C, Soubry A (2017). Male obesity: epigenetic origin and effects in sperm and offspring. Curr Mol Biol Rep.

[9] McPherson NO, Owens JA, Fullston T, Lane M (2015). Preconception diet or exercise intervention in obese fathers normalizes sperm microRNA profile and metabolic syndrome in female offspring. Am J Physiol Endocrinol Metab.

[10] Krout D, Roemmich JN, Bundy A, Garcia RA, Yan L, Claycombe-Larson KJ (2018). Paternal exercise protects mouse offspring from high-fat-diet-induced type 2 diabetes risk by increasing skeletal muscle insulin signaling. J Nutr Biochem.

[11] McPherson NO, Shehadeh H, Fullston T, Zander-Fox DL, Lane M (2019). Dietary Micronutrient Supplementation for 12 Days in Obese Male Mice Restores Sperm Oxidative Stress. Nutrients..

[12] Sutton EF, Gilmore LA, Dunger DB, Heijmans BT, Hivert MF, Ling C (2016). Developmental programming: state-of-the-science and future directions-Summary from a Pennington Biomedical symposium. Obesity.

[13] Gluckman PD, Hanson MA (2004). Living with the past: evolution, development, and patterns of disease. Science..

[14] Barker DJ, Osmond C (1986). Infant mortality, childhood nutrition, and ischaemic heart disease in England and Wales. Lancet..

[15] Hales CN, Barker DJP (1992). Type 2 (non-insulin-dependent) diabetes mellitus: the thrifty phenotype hypothesis. Diabetologia..

[16] Sinclair KD, Watkins AJ (2013). Parental diet, pregnancy outcomes and offspring health: metabolic determinants in developing oocytes and embryos. Reprod Fertil Dev.

[17] Ramaraju GA, Teppala S, Prathigudupu K, Kalagara M, Thota S, Kota M (2018). Association between obesity and sperm quality. Andrologia.

[18] Hammoud AO, Wilde N, Gibson M, Parks A, Carrell DT, Meikle AW (2008). Male obesity and alteration in sperm parameters. Fertil Steril.

[19] Jensen TK, Andersson AM, Jorgensen N, Andersen AG, Carlsen E, Petersen JH (2004). Body mass index in relation to semen quality and reproductive hormones among 1,558 Danish men. Fertil Steril.

[20] Håkonsen LB, Thulstrup AM, Aggerholm AS, Olsen J, Bonde JP, Andersen CY (2011). Does weight loss improve semen quality and reproductive hormones? Results from a cohort of severely obese men. Reprod Health.

[21] Stanford KI, Rasmussen M, Baer LA, Lehnig AC, Rowland LA, White JD (2018). Paternal exercise improves glucose metabolism in adult offspring. Diabetes..

[22] Macdonald AA, Stewart AW, Farquhar CM (2013). Body mass index in relation to semen quality and reproductive hormones in New Zealand men: a cross-sectional study in fertility clinics. Hum Reprod.

[23] Crisóstomo L, Videira RA, Jarak I, Starčević K, Mašek T, Rato L (2020). Diet during early life defines testicular lipid content and sperm quality in adulthood. Am J Physiol-Endocrinol Metab.

[24] Chavarro JE, Toth TL, Wright DL, Meeker JD, Hauser R (2010). Body mass index in relation to semen quality, sperm DNA integrity, and serum reproductive hormone levels among men attending an infertility clinic. Fertil Steril.

[25] Fariello RM, Pariz JR, Spaine DM, Cedenho AP, Bertolla RP, Fraietta R (2012). Association between obesity and alteration of sperm DNA integrity and mitochondrial activity. BJU Int.

[26] Kort HI, Massey JB, Elsner CW, Mitchell-Leef D, Shapiro DB, Witt MA (2006). Impact of body mass index values on sperm quantity and quality. J Androl.

[27] Mir J, Franken D, Andrabi SW, Ashraf M, Rao K (2018). Impact of weight loss on sperm DNA integrity in obese men. Andrologia..

[28] Tunc O, Bakos HW, Tremellen K (2011). Impact of body mass index on seminal oxidative stress. Andrologia..

[29] Palmer NO, Fullston T, Mitchell M, Setchell BP, Lane M (2011). SIRT6 in mouse spermatogenesis is modulated by diet-induced obesity. Reprod Fertil Dev.

[30] Zini A, Boman JM, Belzile E, Ciampi A (2008). Sperm DNA damage is associated with an increased risk of pregnancy loss after IVF and ICSI: systematic review and meta-analysis. Human Reprod.

[31] Ng M, Fleming T, Robinson M, Thomson B, Graetz N, Margono C (2014). Global, regional, and national prevalence of overweight and obesity in children and adults during 1980-2013: a systematic analysis for the Global Burden of Disease Study 2013. Lancet..

[32] Veenendaal M, Painter R, de Rooij S, Bossuyt P, van der Post J, Gluckman P (2013). Transgenerational effects of prenatal exposure to the 1944–45 Dutch famine. BJOG.

[33] Derraik JGB, Pasupathy D, McCowan LME, Poston L, Taylor RS, Simpson NAB (2019). Paternal contributions to large-for-gestational-age term babies: findings from a multicenter prospective cohort study. J Dev Orig Health Dis.

[34] McCowan LM, North RA, Kho EM, Black MA, Chan EH, Dekker GA (2011). Paternal contribution to small for gestational age babies: a multicenter prospective study. Obesity..

[35] Kaati G, Bygren LO, Edvinsson S (2002). Cardiovascular and diabetes mortality determined by nutrition during parents’ and grandparents’ slow growth period. Eur J Hum Genet.

[36] Bygren LO, Kaati G, Edvinsson S (2001). Longevity determined by paternal ancestors’ nutrition during their slow growth period. Acta Biotheoretica.

[37] Pembrey ME, Bygren LO, Kaati G, Edvinsson S, Northstone K, Sjöström M (2006). Sex-specific, male-line transgenerational responses in humans. Eur J Hum Genet.

[38] Bakos HW, Henshaw RC, Mitchell M, Lane M (2011). Paternal body mass index is associated with decreased blastocyst development and reduced live birth rates following assisted reproductive technology. Fertil Steril.

[39] Noor N, Cardenas A, Rifas-Shiman SL, Pan H, Dreyfuss JM, Oken E (2019). Association of periconception paternal body mass index with persistent changes in DNA methylation of offspring in childhood. JAMA Network Open..

[40] Takagi K, Iwama N, Metoki H, Uchikura Y, Matsubara Y, Matsubara K (2019). Paternal height has an impact on birth weight of their offspring in a Japanese population: the Japan Environment and Children’s Study. J Dev Orig Health Dis.

[41] Sørensen TI, Ajslev TA, Ängquist L, Morgen CS, Ciuchi IG, Davey Smith G (2016). Comparison of associations of maternal peri-pregnancy and paternal anthropometrics with child anthropometrics from birth through age 7 y assessed in the Danish National Birth Cohort. Am J Clin Nutr.

[42] Mei H, Guo S, Lu H, Pan Y, Mei W, Zhang B (2018). Impact of parental weight status on children’s body mass index in early life: evidence from a Chinese cohort. BMJ Open.

[43] Zalbahar N, Najman J, McIntyre HD, Mamun A (2017). Parental pre-pregnancy obesity and the risk of offspring weight and body mass index change from childhood to adulthood. Clin Obes.

[44] Cooper R, Hyppönen E, Berry D, Power C (2010). Associations between parental and offspring adiposity up to midlife: the contribution of adult lifestyle factors in the 1958 British Birth Cohort Study. Am J Clin Nutr.

[45] Bjelland M, Lien N, Bergh IH, Grydeland M, Anderssen SA, Klepp KI (2010). Overweight and waist circumference among Norwegian 11-year-olds and associations with reported parental overweight and waist circumference: The HEIA study. Scand J Public Health.

[46] Burke V, Beilin LJ, Simmer K, Oddy WH, Blake KV, Doherty D (2005). Predictors of body mass index and associations with cardiovascular risk factors in Australian children: a prospective cohort study. Int J Obes.

[47] Danielzik S, Czerwinski-Mast M, Langnäse K, Dilba B, Müller MJ (2004). Parental overweight, socioeconomic status and high birth weight are the major determinants of overweight and obesity in 5-7 y-old children: baseline data of the Kiel Obesity Prevention Study (KOPS). Int J Obes Relat Metab Disord.

[48] Burke V, Beilin LJ, Dunbar D (2001). Family lifestyle and parental body mass index as predictors of body mass index in Australian children: a longitudinal study. Int J Obes Relat Metab Disord.

[49] Maffeis C, Micciolo R, Must A, Zaffanello M, Pinelli L (1994). Parental and perinatal factors associated with childhood obesity in north-east Italy. Int J Obes Relat Metab Disord.

[50] Veena SR, Krishnaveni GV, Karat SC, Osmond C, Fall CHD (2013). Testing the fetal overnutrition hypothesis; the relationship of maternal and paternal adiposity to adiposity, insulin resistance and cardiovascular risk factors in Indian children. Public Health Nutr.

[51] Gaillard R, Steegers EAP, Duijts L, Felix JF, Hofman A, Franco OH (2014). Childhood cardiometabolic outcomes of maternal obesity during pregnancy. Hypertension..

[52] McCarthy K, Ye YL, Yuan S, He QQ (2015). Parental weight status and offspring cardiovascular disease risks: a cross-sectional study of Chinese children. Prev Chronic Dis.

[53] Labayen I, Ruiz JR, Ortega FB, Loit HM, Harro J, Veidebaum T (2010). Intergenerational cardiovascular disease risk factors involve both maternal and paternal BMI. Diabetes Care.

[54] Yang Z, Li Y, Dong B, Gao D, Wen B, Ma J (2020). Relationship between parental overweight and obesity and childhood metabolic syndrome in their offspring: result from a cross-sectional analysis of parent-offspring trios in China. BMJ Open.

[55] Jääskeläinen A, Pussinen J, Nuutinen O, Schwab U, Pirkola J, Kolehmainen M (2011). Intergenerational transmission of overweight among Finnish adolescents and their parents: a 16-year follow-up study. Int J Obes.

[56] Zalbahar N, Najman J, McIntrye HD, Mamun A (2016). Parental pre-pregnancy BMI influences on offspring BMI and waist circumference at 21 years. Aust N Z J Public Health.

[57] Power C, Pouliou T, Li L, Cooper R, Hyppönen E (2011). Parental and offspring adiposity associations: insights from the 1958 British birth cohort. Ann Hum Biol.

[58] Lake JK, Power C, Cole TJ (1997). Child to adult body mass index in the 1958 British birth cohort: associations with parental obesity. Arch Dis Child.

[59] Murugappan G, Li S, Leonard S, Winnm VD, Druzin M, Eisenberg ML (2021). Association of preconception paternal health and adverse maternal outcomes among healthy mothers. Am J Obstet Gynecol MFM..

[60] Kasman AM, Zhang CA, Li S, Stevenson DK, Shaw GM, Eisenberg ML (2020). Association of preconception paternal health on perinatal outcomes: analysis of U.S. claims data. Fertil Steril.

[61] Magnus MC, Olsen SF, Granstrom C, Lund-Blix NA, Svensson J, Johannesen J (2018). Paternal and maternal obesity but not gestational weight gain is associated with type 1 diabetes. Int J Epidemiol.

[62] Santos Ferreira DL, Williams DM, Kangas AJ, Soininen P, Ala-Korpela M, Smith GD (2017). Association of pre-pregnancy body mass index with offspring metabolic profile: Analyses of 3 European prospective birth cohorts. PLoS Med.

[63] Chen YP, Xiao XM, Li J, Reichetzeder C, Wang ZN, Hocher B (2012). Paternal body mass index (BMI) is associated with offspring intrauterine growth in a gender dependent manner. PLoS One.

[64] Soubry A, Schildkraut JM, Murtha A, Wang F, Huang Z, Bernal A (2013). Paternal obesity is associated with IGF2 hypomethylation in newborns: results from a Newborn Epigenetics Study (NEST) cohort. BMC Med.

[65] Soubry A, Murphy SK, Wang F, Huang Z, Vidal AC, Fuemmeler BF (2015). Newborns of obese parents have altered DNA methylation patterns at imprinted genes. Int J Obes.

[66] Baker J, Liu JP, Robertson EJ, Efstratiadis A (1993). Role of insulin-like growth factors in embryonic and postnatal growth. Cell..

[67] Takahashi M, Kamei Y, Ezaki O (2005). Mest/Peg1 imprinted gene enlarges adipocytes and is a marker of adipocyte size. Am J Physiol Endocrinol Metab.

[68] Vrang N, Meyre D, Froguel P, Jelsing J, Tang-Christensen M, Vatin V (2010). The imprinted gene neuronatin is regulated by metabolic status and associated with obesity. Obesity.

[69] Binder NK, Hannan NJ, Gardner DK (2012). Paternal diet-induced obesity retards early mouse embryo development, mitochondrial activity and pregnancy health. PLoS One.

[70] Binder NK, Mitchell M, Gardner DK (2012). Parental diet-induced obesity leads to retarded early mouse embryo development and altered carbohydrate utilisation by the blastocyst. Reprod Fertil Dev.

[71] Binder NK, Beard SA, Kaitu’u-Lino TJ, Tong S, Hannan NJ, Gardner DK (2015). Paternal obesity in a rodent model affects placental gene expression in a sex-specific manner. Reproduction..

[72] Martinvalet D, Zhu P, Lieberman J (2005). Granzyme A induces caspase-independent mitochondrial damage, a required first step for apoptosis. Immunity..

[73] Liu H, Tang Y, Liu X, Zhou Q, Xiao X, Lan F (2014). 14-3-3 tau (YWHAQ) gene promoter hypermethylation in human placenta of preeclampsia. Placenta..

[74] Hedegger K, Philippou-Massier J, Krebs S, Blum H, Kunzelmann S, Förstemann K (2020). Sex-specific programming effects of parental obesity in pre-implantation embryonic development. Int J Obes.

[75] Ng SF, Lin RC, Laybutt DR, Barres R, Owens JA, Morris MJ (2010). Chronic high-fat diet in fathers programs beta-cell dysfunction in female rat offspring. Nature..

[76] Ng SF, Lin RC, Maloney CA, Youngson NA, Owens JA, Morris MJ (2014). Paternal high-fat diet consumption induces common changes in the transcriptomes of retroperitoneal adipose and pancreatic islet tissues in female rat offspring. Faseb J.

[77] Chowdhury SS, Lecomte V, Erlich JH, Maloney CA, Morris MJ (2016). Paternal high fat diet in rats leads to renal accumulation of lipid and tubular changes in adult offspring. Nutrients..

[78] Lecomte V, Maloney CA, Wang KW, Morris MJ (2017). Effects of paternal obesity on growth and adiposity of male rat offspring. Am J Physiol Endocrinol Metab.

[79] Crisóstomo L, Jarak I, Rato LP, Raposo JF, Batterham RL, Oliveira PF (2021). Inheritable testicular metabolic memory of high-fat diet causes transgenerational sperm defects in mice. Sci Rep.

[80] Fullston T, Ohlsson Teague EM, Palmer NO, DeBlasio MJ, Mitchell M, Corbett M (2013). Paternal obesity initiates metabolic disturbances in two generations of mice with incomplete penetrance to the F2 generation and alters the transcriptional profile of testis and sperm microRNA content. Faseb J.

[81] Ornellas F, Souza-Mello V, Mandarim-de-Lacerda CA, Aguila MB (2015). Programming of obesity and comorbidities in the progeny: lessons from a model of diet-induced obese parents. PLoS One.

[82] Masuyama H, Mitsui T, Eguchi T, Tamada S, Hiramatsu Y (2016). The effects of paternal high-fat diet exposure on offspring metabolism with epigenetic changes in the mouse adiponectin and leptin gene promoters. Am J Physiol-Endocrinol Metab.

[83] Sanchez-Garrido MA, Ruiz-Pino F, Velasco I, Barroso A, Fernandois D, Heras V (2018). Intergenerational Influence of Paternal Obesity on Metabolic and Reproductive Health Parameters of the Offspring: Male-Preferential Impact and Involvement of Kiss1-Mediated Pathways. Endocrinology..

[84] Chambers TJ, Morgan MD, Heger AH, Sharpe RM, Drake AJ (2016). High-fat diet disrupts metabolism in two generations of rats in a parent-of-origin specific manner. Sci Rep.

[85] Fullston T, McPherson NO, Owens JA, Kang WX, Sandeman LY, Lane M (2015). Paternal obesity induces metabolic and sperm disturbances in male offspring that are exacerbated by their exposure to an “obesogenic” diet. Physiol Rep.

[86] Grandjean V, Fourré S, De Abreu DA, Derieppe MA, Remy JJ, Rassoulzadegan M (2015). RNA-mediated paternal heredity of diet-induced obesity and metabolic disorders. Sci Rep.

[87] Terashima M, Barbour S, Ren J, Yu W, Han Y, Muegge K (2015). Effect of high fat diet on paternal sperm histone distribution and male offspring liver gene expression. Epigenetics..

[88] de Castro Barbosa T, Ingerslev LR, Alm PS, Versteyhe S, Massart J, Rasmussen M (2016). High-fat diet reprograms the epigenome of rat spermatozoa and transgenerationally affects metabolism of the offspring. Mol Metab.

[89] Cropley JE, Eaton SA, Aiken A, Young PE, Giannoulatou E, Ho JWK (2016). Male-lineage transmission of an acquired metabolic phenotype induced by grand-paternal obesity. Molecular Metab.

[90] Fullston T, Palmer NO, Owens JA, Mitchell M, Bakos HW, Lane M (2012). Diet-induced paternal obesity in the absence of diabetes diminishes the reproductive health of two subsequent generations of mice. Hum Reprod.

[91] Fullston T, Shehadeh H, Sandeman LY, Kang WX, Wu LL, Robker RL (2015). Female offspring sired by diet induced obese male mice display impaired blastocyst development with molecular alterations to their ovaries, oocytes and cumulus cells. J Assist Reprod Genet.

[92] Oshio LT, Andreazzi AE, Lopes JF, Sá JP, Bolotari M, Costa VMG (2020). A paternal hypercaloric diet affects the metabolism and fertility of F1 and F2 Wistar rat generations. J Dev Orig Health Dis.

[93] McPherson NO, Fullston T, Aitken RJ, Lane M (2014). Paternal obesity, interventions, and mechanistic pathways to impaired health in offspring. Ann Nutr Metab.

[94] Bromfield JJ, Schjenken JE, Chin PY, Care AS, Jasper MJ, Robertson SA (2014). Maternal tract factors contribute to paternal seminal fluid impact on metabolic phenotype in offspring. Proc Natl Acad Sci USA.

[95] Eisenberg ML, Kim S, Chen Z, Sundaram R, Schisterman EF, Buck, Louis GM (2014). The relationship between male BMI and waist circumference on semen quality: data from the LIFE study. Hum Reprod.

[96] Martini AC, Tissera A, Estofán D, Molina RI, Mangeaud A, de Cuneo MF (2010). Overweight and seminal quality: a study of 794 patients. Fertil Steril.

[97] Lotti F, Corona G, Colpi GM, Filimberti E, Degli Innocenti S, Mancini M (2011). Elevated body mass index correlates with higher seminal plasma interleukin 8 levels and ultrasonographic abnormalities of the prostate in men attending an andrology clinic for infertility. J Endocrinol Investig.

[98] Thomas S, Kratzsch D, Schaab M, Scholz M, Grunewald S, Thiery J (2013). Seminal plasma adipokine levels are correlated with functional characteristics of spermatozoa. Fertil Steril.

[99] Binder NK, Sheedy JR, Hannan NJ, Gardner DK (2015). Male obesity is associated with changed spermatozoa Cox4i1 mRNA level and altered seminal vesicle fluid composition in a mouse model. Mol Hum Reprod.

[100] Schjenken JE, Moldenhauer LM, Sharkey DJ, Chan HY, Chin PY, Fullston T (2021). High-fat Diet Alters Male Seminal Plasma Composition to Impair Female Immune Adaptation for Pregnancy in Mice. Endocrinology..

[101] Hur SS, Cropley JE, Suter CM (2017). Paternal epigenetic programming: evolving metabolic disease risk. J Mol Endocrinol.

[102] Wei Y, Yang CR, Wei YP, Zhao ZA, Hou Y, Schatten H (2014). Paternally induced transgenerational inheritance of susceptibility to diabetes in mammals. Proc Natl Acad Sci USA.

[103] Dupont C, Kappeler L, Saget S, Grandjean V, Lévy R (2019). Role of miRNA in the Transmission of Metabolic Diseases Associated With Paternal Diet-Induced Obesity. Front Genet.

[104] Zhang Y, Shi J, Rassoulzadegan M, Tuorto F, Chen Q (2019). Sperm RNA code programmes the metabolic health of offspring. Nat Rev Endocrinol.

[105] Yan M, Zhai Q (2016). Sperm tsRNAs and acquired metabolic disorders. J Endocrinol.

[106] Lane M, McPherson NO, Fullston T, Spillane M, Sandeman L, Kang WX (2014). Oxidative stress in mouse sperm impairs embryo development, fetal growth and alters adiposity and glucose regulation in female offspring. PLoS One.

[107] Aitken J, Fisher H (1994). Reactive oxygen species generation and human spermatozoa: the balance of benefit and risk. Bioessays..

[108] Aitken RJ, Baker MA (2002). Reactive oxygen species generation by human spermatozoa: a continuing enigma. Int J Androl.

[109] Menezo YJ, Silvestris E, Dale B, Elder K (2016). Oxidative stress and alterations in DNA methylation: two sides of the same coin in reproduction. Reprod Biomed Online.

[110] Xavier MJ, Roman SD, Aitken RJ, Nixon B (2019). Transgenerational inheritance: how impacts to the epigenetic and genetic information of parents affect offspring health. Human Reproduction Update.

[111] Lewis SE, Aitken RJ (2005). DNA damage to spermatozoa has impacts on fertilization and pregnancy. Cell Tissue Res.

[112] Borini A, Tarozzi N, Bizzaro D, Bonu MA, Fava L, Flamigni C (2006). Sperm DNA fragmentation: paternal effect on early post-implantation embryo development in ART. Hum Reprod.

[113] Simon L, Murphy K, Shamsi MB, Liu L, Emery B, Aston KI (2014). Paternal influence of sperm DNA integrity on early embryonic development. Hum Reprod.

[114] Robinson L, Gallos ID, Conner SJ, Rajkhowa M, Miller D, Lewis S (2012). The effect of sperm DNA fragmentation on miscarriage rates: a systematic review and meta-analysis. Hum Reprod.

[115] Osman A, Alsomait H, Seshadri S, El-Toukhy T, Khalaf Y (2015). The effect of sperm DNA fragmentation on live birth rate after IVF or ICSI: a systematic review and meta-analysis. Reprod Biomed Online.

[116] Kodama H, Yamaguchi R, Fukuda J, Kasai H, Tanaka T (1997). Increased oxidative deoxyribonucleic acid damage in the spermatozoa of infertile male patients. Fertil Steril.

[117] Barroso G, Morshedi M, Oehninger S (2000). Analysis of DNA fragmentation, plasma membrane translocation of phosphatidylserine and oxidative stress in human spermatozoa. Hum Reprod.

[118] Kemal Duru N, Morshedi M, Oehninger S (2000). Effects of hydrogen peroxide on DNA and plasma membrane integrity of human spermatozoa. Fertil Steril.

[119] McPherson NO, Fullston T, Kang WX, Sandeman LY, Corbett MA, Owens JA (2016). Paternal under-nutrition programs metabolic syndrome in offspring which can be reversed by antioxidant/vitamin food fortification in fathers. Sci Rep.

[120] Dulloo AG, Jacquet J, Seydoux J, Montani JP (2006). The thrifty ‘catch-up fat’ phenotype: its impact on insulin sensitivity during growth trajectories to obesity and metabolic syndrome. Int J Obes.

[121] Perrard M-H, Sereni N, Schluth-Bolard C, Blondet A, d′Estaing SG, Plotton I (2016). Complete Human and Rat Ex Vivo Spermatogenesis from Fresh or Frozen Testicular Tissue1. Biol Reprod.

[122] Bray GA, Heisel WE, Afshin A, Jensen MD, Dietz WH, Long M (2018). The Science of Obesity Management: An Endocrine Society Scientific Statement. Endocr Rev.

[123] Heymsfield SB, Wadden TA (2017). Mechanisms, pathophysiology, and management of obesity. N. Engl J Med.

[124] Adams TD, Davidson LE, Litwin SE, Kim J, Kolotkin RL, Nanjee MN (2017). Weight and metabolic outcomes 12 years after gastric bypass. N. Engl J Med.

[125] Samavat J, Cantini G, Lotti F, Di Franco A, Tamburrino L, Degl’Innocenti S (2018). Massive Weight Loss Obtained by Bariatric Surgery Affects Semen Quality in Morbid Male Obesity: a Preliminary Prospective Double-Armed Study. Obes Surg.

[126] Lee Y, Dang JT, Switzer N, Yu J, Tian C, Birch DW (2019). Impact oF Bariatric Surgery on Male Sex Hormones and Sperm Quality: A Systematic Review and Meta-analysis. Obes Surg.

[127] Denham J, O’Brien BJ, Harvey JT, Charchar FJ (2015). Genome-wide sperm DNA methylation changes after 3 months of exercise training in humans. Epigenomics..

[128] Donkin I, Versteyhe S, Ingerslev LR, Qian K, Mechta M, Nordkap L (2016). Obesity and Bariatric Surgery Drive Epigenetic Variation of Spermatozoa in Humans. Cell Metab.

[129] Ingerslev LR, Donkin I, Fabre O, Versteyhe S, Mechta M, Pattamaprapanont P (2018). Endurance training remodels sperm-borne small RNA expression and methylation at neurological gene hotspots. Clin Epigenet.

[130] Donkin I, Barrès R (2018). Sperm epigenetics and influence of environmental factors. Mol Metab.

[131] McPherson NO, Bakos HW, Owens JA, Setchell BP, Lane M (2013). Improving metabolic health in obese male mice via diet and exercise restores embryo development and fetal growth. PLoS One.

[132] McPherson NO, Lane M, Sandeman L, Owens JA, Fullston T (2017). An exercise-only intervention in obese fathers restores glucose and insulin regulation in conjunction with the rescue of pancreatic islet cell morphology and MicroRNA expression in male offspring. Nutrients..

[133] Zheng J, Alves-Wagner AB, Stanford KI, Prince NB, So K, Mul JD (2020). Maternal and paternal exercise regulate offspring metabolic health and beta cell phenotype. BMJ Open Diabetes Res Care.

[134] Batista RO, Budu A, Alves-Silva T, Arakaki AM, Gregnani MFS, Rodrigues Húngaro TG (2020). Paternal exercise protects against liver steatosis in the male offspring of mice submitted to high fat diet. Life Sci.

[135] Bailey RL, West KP, Black RE (2015). The epidemiology of global micronutrient deficiencies. Ann Nutr Metab.

[136] Kimmons JE, Blanck HM, Tohill BC, Zhang J, Khan LK (2006). Associations between body mass index and the prevalence of low micronutrient levels among US adults. MedGenMed.

[137] Xanthakos SA (2009). Nutritional deficiencies in obesity and after bariatric surgery. Pediatr Clin North Am.

[138] Aasheim ET, Hofso D, Hjelmesaeth J, Birkeland KI, Bohmer T (2008). Vitamin status in morbidly obese patients: a cross-sectional study. Am J Clin Nutr.

[139] Ernst B, Thurnheer M, Schmid SM, Schultes B (2009). Evidence for the necessity to systematically assess micronutrient status prior to bariatric surgery. Obes Surg.

[140] Hosseini B, Saedisomeolia A, Allman-Farinelli M (2017). Association between antioxidant intake/status and obesity: a systematic review of observational studies. Biol Trace Elem Res.

[141] McKay J, Ho S, Jane M, Pal S (2020). Overweight & obese Australian adults and micronutrient deficiency. BMC Nutr.

[142] Huang Q, Wang L, Jiang H, Wang H, Zhang B, Zhang J (2020). Intra-Individual Double Burden of Malnutrition among Adults in China: evidence from the China Health and Nutrition Survey 2015. Nutrients..

[143] Ebisch IM, Thomas CM, Peters WH, Braat DD, Steegers-Theunissen RP (2007). The importance of folate, zinc and antioxidants in the pathogenesis and prevention of subfertility. Hum Reprod Update.

[144] Ebisch IM, Pierik FH, Fh DEJ, Thomas CM, Steegers-Theunissen RP (2006). Does folic acid and zinc sulphate intervention affect endocrine parameters and sperm characteristics in men?. Int J Androl.

[145] Irani M, Amirian M, Sadeghi R, Lez JL, Latifnejad Roudsari R (2017). The Effect of Folate and Folate Plus Zinc Supplementation on Endocrine Parameters and Sperm Characteristics in Sub-Fertile Men: A Systematic Review and Meta-Analysis. Urol J..

[146] Barik G, Chaturvedula L, Bobby Z (2019). Role of Oxidative Stress and Antioxidants in Male Infertility: An Interventional Study. J Hum Reprod Sci.

[147] Zhao J, Dong X, Hu X, Long Z, Wang L, Liu Q (2016). Zinc levels in seminal plasma and their correlation with male infertility: a systematic review and meta-analysis. Sci Rep.

[148] Tunc O, Thompson J, Tremellen K (2009). Improvement in sperm DNA quality using an oral antioxidant therapy. Reprod Biomed Online.

[149] Jannatifar R, Parivar K, Roodbari NH, Nasr-Esfahani MH (2019). Effects of N-acetyl-cysteine supplementation on sperm quality, chromatin integrity and level of oxidative stress in infertile men. Reprod Biol Endocrinol.

[150] Gharagozloo P, Gutiérrez-Adán A, Champroux A, Noblanc A, Kocer A, Calle A (2016). A novel antioxidant formulation designed to treat male infertility associated with oxidative stress: promising preclinical evidence from animal models. Hum Reprod.

[151] Khatiwada S, Lecomte V, Morris M, Maloney C. A Novel Micronutrient Supplement to Preserve Male Metabolic Health and Prevent Fatty Liver (P21-007-19). Curr Dev Nutr. 2019;3. 10.1093/cdn/nzz041.P21-007-19.

[152] Billah MM, Khatiwada S, Lecomte V, Morris M, Maloney C (2020). Effect of high fat diet induced paternal obesity andmicronutrient intervention on offspring testicular antioxidant capacity. 36th Virtual Annual Meeting of the EuropeanSociety of Human Reproduction and Embryology. Hum Repro.

[153] Billah MM, Khatiwada S, Lecomte V, Morris M, Maloney C. A Novel Micronutrient Supplement to Mitigate the Transgenerational Effects of Paternal Obesity on Body Composition of Male Offspring (P11-138-19). Curr Dev Nutr. 2019;3. 10.1093/cdn/nzz048.P11-138-19.

[154] Chleilat F, Schick A, Deleemans JM, Reimer RA (2021). Paternal methyl donor supplementation in rats improves fertility, physiological outcomes, gut microbial signatures and epigenetic markers altered by high fat/high sucrose diet. Int J Mol Sci.

[155] Oakberg EF (1957). Duration of spermatogenesis in the mouse. Nature..

[156] McPherson NO, Lane M (2020). Metformin treatment of high-fat diet-fed obese male mice restores sperm function and fetal growth, without requiring weight loss. Asian J Androl.

[157] Santos-Silva JC, Ribeiro RA, Vettorazzi JF, Irles E, Rickli S, Borck PC (2015). Taurine supplementation ameliorates glucose homeostasis, prevents insulin and glucagon hypersecretion, and controls β, α, and δ-cell masses in genetic obese mice. Amino Acids.

[158] Ribeiro RA, Bonfleur ML, Batista TM, Borck PC, Carneiro EM (2018). Regulation of glucose and lipid metabolism by the pancreatic and extra-pancreatic actions of taurine. Amino Acids.

[159] Yang J, Wu G, Feng Y, Lv Q, Lin S, Hu J (2010). Effects of taurine on male reproduction in rats of different ages. J Biomed Sci.

[160] Freitas IN, Dos Reis Araujo T, Vettorazzi JF, Magalhaes EA, Carneiro EM, Bonfleur ML (2019). Taurine supplementation in high-fat diet fed male mice attenuates endocrine pancreatic dysfunction in their male offspring. Amino Acids.

